# Prospects for sustainability of pig production in relation to climate change and novel feed resources

**DOI:** 10.1002/jsfa.10338

**Published:** 2020-03-14

**Authors:** Wendy M Rauw, Lotta Rydhmer, Ilias Kyriazakis, Margareth Øverland, Hélène Gilbert, Jack CM Dekkers, Susanne Hermesch, Alban Bouquet, Emilio Gómez Izquierdo, Isabelle Louveau, Luis Gomez‐Raya

**Affiliations:** ^1^ Departamento de Mejora Genética Animal INIA Madrid Spain; ^2^ Department of Animal Breeding and Genetics Swedish University of Agricultural Sciences Uppsala Sweden; ^3^ Institute for Global Food Security, Queen's University, Belfast, UK; ^4^ Department of Animal and Aquacultural Sciences Norwegian University of Life Sciences Ås Norway; ^5^ GenPhySE, Université de Toulouse, INRAE Castanet Tolosan France; ^6^ Department of Animal Science Iowa State University Ames IA USA; ^7^ Animal Genetics and Breeding Unit (a joint venture of NSW Department of PrimaryIndustries and University of New England), University of New England Armidale Australia; ^8^ IFIP‐Institut du Porc Le Rheu, Cedex France; ^9^ Centro de Pruebas de Porcino, ITACyL Hontalbilla Spain; ^10^ PEGASE, INRAE, Agrocampus Ouest Saint‐Gilles France

**Keywords:** sustainable agriculture, animal robustness, climate change, local feed resources, pig production

## Abstract

Pig production systems provide multiple benefits to humans. However, the global increase in meat consumption has profound consequences for our earth. This perspective describes two alternative scenarios for improving the sustainability of future pig production systems. The first scenario is a high input–high output system based on sustainable intensification, maximizing animal protein production efficiency on a limited land surface at the same time as minimizing environmental impacts. The second scenario is a reduced input–reduced output system based on selecting animals that are more robust to climate change and are better adapted to transform low quality feed (local feeds, feedstuff co‐products, food waste) into meat. However, in contrast to the first scenario, the latter scenario results in reduced predicted yields, reduced production efficiency and possibly increased costs to the consumer. National evaluation of the availability of local feed and feedstuff co‐product alternatives, determination of limits to feed sourced from international markets, available land for crop and livestock production, desired production levels, and a willingness to politically enforce policies through subsidies and/or penalties are some of the considerations to combine these two scenarios. Given future novel sustainable alternatives to livestock animal protein, it may become reasonable to move towards an added general premium price on ‘protein from livestock animals’ to the benefit of promoting higher incomes to farmers at the same time as covering the extra costs of, politically enforced, welfare of livestock animals in sustainable production systems. © 2020 The Authors. *Journal of The Science of Food and Agriculture* published by John Wiley & Sons Ltd on behalf of Society of Chemical Industry.

## INTRODUCTION

**Figure 1:**
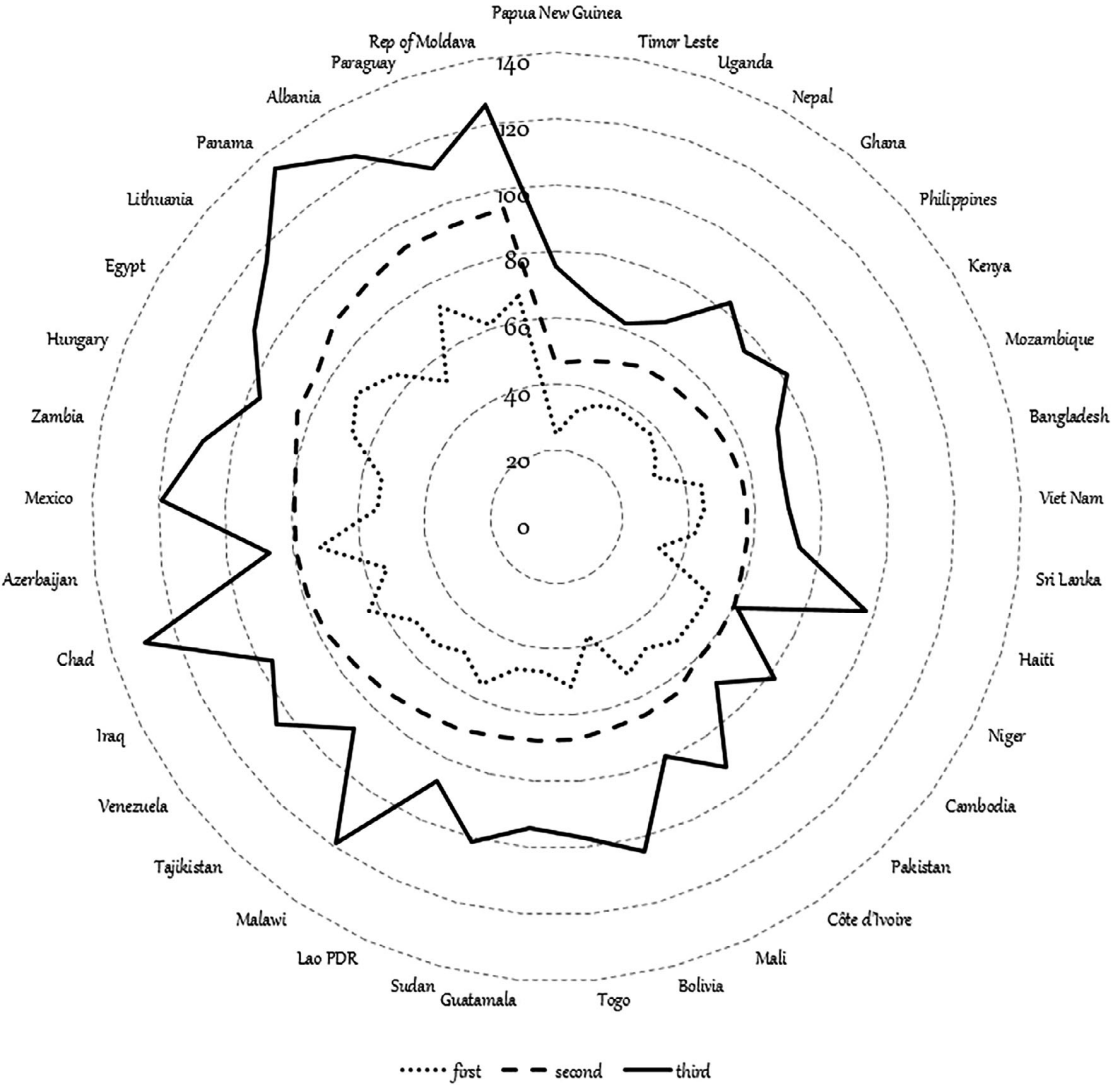
Protein consumption (g capita^–1^ day^–1^) in 37 countries by income tercile; based on data downloaded from the FAO.^5^

**Figure 2:**
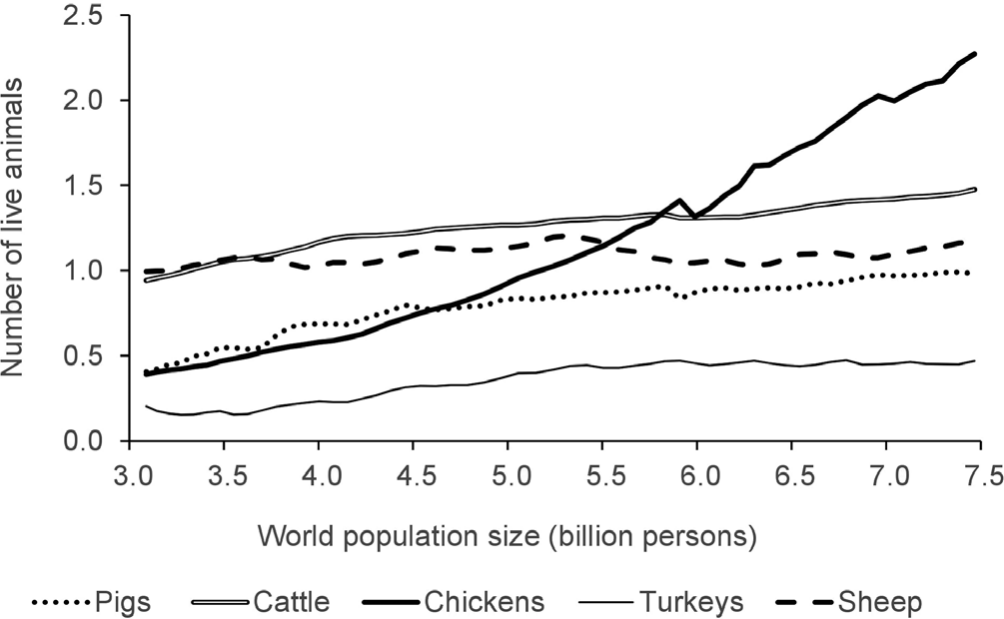
Increase in the world's animal populations from different livestock and poultry species in response to the increase in the world's human population size (between 1961 and 2013; based on data downloaded from the FAO.^11^). Pigs, cattle, turkeys and sheep × 1000 000 000; Chickens × 10 000 000 000.

The United Nations Population Division^1^ projects that the human population may rise to almost 11 billion people by 2100. Almost 90% of the population will live in less developed regions. In particular, nine countries will be responsible for more than half of the projected population growth: India, Nigeria, Pakistan, Congo, Ethiopia, Tanzania, Indonesia, Egypt and the USA.^2^ Parallel to overall population growth, there is evidence for an increase in meat production and consumption. From 1961 to 2013, average annual meat consumption rose worldwide from 23.1 to 43.2 kg per person: between 14 kg in the least developed countries to over 81 kg in the European Union and 115 kg in the USA and Australia, achieving average consumption levels that exceed needs in the most developed countries.^3^, ^4^ Even though the number of undernourished people is estimated to have reached 821 million in 2017,^5^ the purchasing power of the developing world increased significantly in the 2000s, and to such a rate that the aggregate economic weight of developing and emerging economies has surpassed that of the countries that currently make up the advanced world.^6^ Figure 1 shows an increase in protein consumption (g/capita/d) in 37 countries by income tercile^5^: as income grows, so does expenditure on livestock products.^7^ Protein consumption increased between 50% and 200% when income increased from the first to the third tercile (Fig. 1); this variation in consumption increase can be explained by differences in initial intake levels and the relative place of meat in protein intake in each country.^3^ The increase in population size and consumption *per capita* propelled, what Delgado^8^ called, ‘the livestock revolution’. The increase in the world production of meat from different livestock species in response to the increase in the world's human population size is particularly pronounced for broiler and pig meat.^9^ So much so that Thomas *et al*.^10^ exclaimed: ‘We are living on the planet of the chickens. The broiler (meat) chicken now outweighs all wild birds together by three to one’. Although breed choice and selection practices have improved production yield per animal, this increased demand for animal products has indeed resulted in an unprecedented increase in the world's livestock populations (1961 to 2016) (Fig. 2): according to data available to the Food and Agriculture Organization (FAO),^11^ in 2017, each 100 persons shared this world with approximately 13 pigs, 20 cattle, 303 chickens, 6 turkeys 14 goats and 16 sheep.

Livestock farming systems provide a range of benefits, including the provision of protein‐rich food from edible resources that contribute to food security, employment and rural economies, carbon storage and flood control by grasslands, landscape aesthetic value, recreation and tourism potential, capital stock and draught power in many developing countries, cultural identity, and social services all around the world.^12^, ^13^ Meat consumption contributes to the supply of energy, protein, and important micronutrients (e.g. long‐chain n‐3 fatty acids, copper, iron, iodine, manganese, selenium, zinc, B‐vitamins) in the human food chain.^14^ However, despite these multiple benefits of meat production, the increase in the number of livestock animals directly challenges sustainability of animal production because it results in profound consequences for our earth: in 2004, the WorldWatch Institute^15^ concluded: ‘The human appetite for animal flesh is a driving force behind virtually every major category of environmental damage now threatening the human future – deforestation, erosion, fresh water scarcity, air and water pollution, climate change, biodiversity loss, social injustice, the destabilization of communities and the spread of disease’. ‘Sustainability’ was first debated at the 1972 United Nations Conference on the Human Environment held in Stockholm, Sweden, where it was defined as ‘an economy in equilibrium with its basic ecological support system’; that is, referring to the balance between population growth and activities that draw on the earth's finite natural resources.^16^ Following the meeting, the concept of sustainability has been shaped by science, popular movements, and formal global networks into very different conceptualizations that may focus on different dimensions of sustainability.^17^ The most quoted definition of sustainable development is taken from the 1987 report of the World Commission on Environment and Development: ‘Sustainable development is development that meets the needs of the present without compromising the ability of future generations to meet their own needs’. This definition is not a blueprint of sustainability because it covers economic, environmental, as well as social systems that differ widely among countries.^18^ Following these three pillars of sustainability, the Europen Union (EU) defined the aims of sustainable agriculture to ensure economic viability, conserve natural resources, deliver ecosystem services, manage the countryside, improve the quality of life in farming areas, to insure animal welfare, and to produce safe and healthy food.^19^ The EU goal for animal production in particular is to enhance competitiveness and the economic viability of animal production systems; improve the adaptation of livestock to vulnerable diseases and increasingly extreme weather patterns associated with climate change; and solve issues related to diet and health, ammonia and air quality, greenhouse gas emissions and climate change, degradation of natural resources such as nitrates emissions and water, soil and biodiversity, global food security, global trade, and animal wellbeing.^20^ It follows that negative implications of livestock production can be mitigated through improvement of sustainability of farming procedures. Sustainability of livestock production can be improved with an increase in production efficiency per animal by means of genetic selection, and precision livestock farming.^21^ However, technification of livestock systems is not available to the entire livestock sector. Instead, modern livestock animals are often challenged to perform in a wide variety of suboptimal environmental conditions, regarding climate, housing facilities, social environment, disease pressure, and differences in feed quality and composition.^22^, ^23^ For example, a shift towards warmer climates requires animals that are more robust to heat stress.^24^ In addition, and opposite to the concept of precision livestock farming, sustainability is also increased with a shift from reliance on optimally formulated feeds based on feed grains and imported feedstuffs to local feeds and feedstuff co‐products of sub‐optimal quality.^25^ According to Dagevos and Voordouw,^26^ sustainability measures should also include strategies to reduce meat consumption and to encourage more sustainable eating practices.

It is the aim of the present perspective to present an overview of two scenarios: agricultural intensification (high input–high output systems) *versus* improved robustness to suboptimal conditions (reduced input–reduced output systems). First, we discuss the importance of sustainability measures to mitigate negative implications of an increase in the number of livestock animals to our earth. We then describe the concept of precision livestock farming as a scenario to improve sustainability of livestock farming. We follow with a description of increased adaptation to climate change and local feed resources as an alternative scenario to improve sustainability. Finally, we discuss the economic viability of these two scenarios. In Europe, the population is expected to increase very little, and the demand for animal products is unlikely to increase; however, European diets are on average high in animal products.^27^ This perspective emphasizes European livestock production systems.

## SUSTAINABILITY OF LIVESTOCK PRODUCTION

### Land, water and energy inputs

Although the increase in demand for animal products over the last decades has been largely met by intensive livestock production,^28^ increased demand must likely result in an increase of land dedicated to grow and feed livestock. Because the total arable global surface is limited, this seriously challenges sustainability of production: currently, livestock production, including grazing land and land dedicated to feedcrop production, already accounts for approximately 70% of all agricultural land and 30% of the total land surface of the planet.^29^ Release of the amount of carbon held in trees to the atmosphere with the clearing of natural vegetation for agricultural production, which is 20 to 50 times higher in forests than in cleared lands, contributes to the greenhouse effect and global warming.^30^ According to Houghton,^31^ tropical deforestation, in particular in Brazil, India and Indonesia, is estimated to have released 1–2 PgC (petagrams of carbon) per year during the 1990s and is predicted to release another 85–130 PgC over the next 100 years. The livestock sector accounts for 8 % of global human water use, mostly for the irrigation of feedcrops.^29^ Pimentel *et al*.^32^ estimate that the liters of water needed per kg product ranges from 3500 in broiler chickens, 6000 in pigs, 43 000 in feedlot beef and 120 000–200 000 for beef produced on open rangeland, as opposed to 650 for corn, 900 for wheat, 1000 for cereal grain, 1600 for rice and 2000 for soybean. According to estimates by Hoekstra and Chapagain,^33^ the water needed to produce chicken meat, pork and beef is 3900, 4900, and 15 500 m^3^ ton^–1^, respectively, compared with 900 m^3^ ton^–1^ for maize, 1300 m^3^ ton^–1^ for wheat and 3000 m^3^ ton^–1^ for husked rice. A recent study by Mekkonen *et al*.^34^ showed that several factors, including larger livestock output per head, lower feed requirements per head and larger yields of feed crops, have resulted in improved water productivity (i.e. the ratio of the product output per animal to its water footprint) of meat and milk products between 1960 and 2016. However, they warn that the livestock sector still consumes large amounts of water, contributing to the competition over scarce freshwater resources. Furthermore, livestock production requires energy. In the light of the 1970s oil crisis, Pimentel *et al*.^35^ warned that the use of high energy production technology to sustain green revolution agriculture might have a significant impact on agriculture as an industry and a way of life when conventional energy resources become scarce and expensive. According to Pimentel,^36^ kcal fossil energy inputs per kcal of animal protein produced increases from 4:1 in broilers, to 10:1 in turkeys, 14:1 in dairy cows, 14:1 in pigs, 20:1 in grass‐fed beef cattle, 39:1 in laying hens, 40:1 in grain‐ and forage‐fed beef cattle, to 57:1 in lamb. The major fossil energy inputs come from fertilizers, farm machinery, fuel, irrigation and pesticides for grain and forage production.^37^

### Emissions and wastes

A larger livestock population results in larger amounts of emissions and wastes. The livestock supply chain is estimated to be responsible for the emission of 44% of anthropogenic methane (mostly from enteric fermentation by ruminants), 53% of anthropogenic nitrous oxide (mostly from manure) and 5% of anthropogenic carbon dioxide, contributing to global warming, and acidification and eutrophication of ecosystems.^38^ As quantified by Schiehorn *et al*.,^39^ with the collapse of the Soviet Union in 1991, emissions in carbon dioxide equivalents dropped by 7.61 Gt, mainly resulting from a decrease in beef production, increase in beef imports and carbon sequestration in soils on abandoned cropland. According to Campbell *et al*.,^40^ of the nine recognized planetary boundaries that define a safe operating space for humanity, agriculture is the major driver of full transgression of ‘biosphere integrity’ and ‘biogeochemical flows’, is a significant contributor to ‘climate change’, puts ‘land‐system change’ and ‘freshwater use’ at increasing risk of transgression, and threatens the planetary boundaries of ‘ocean acidification’, ‘stratospheric ozone depletion’, ‘atmospheric aerosol loading’ and ‘introduction of novel entities’ that are still in the safe zone.

### Reducing meat consumption

Because of the impact of agriculture in particular, the need for a synergistic combination of changing Western diets to (more) plant‐based, less intensive food types (i.e. a reduction of meat intake), improvements in technologies and management, and reductions in food loss and waste are emphasized.^41^, ^42^, ^43^ For example, Raphaely and Marinova^44^ note: ‘Flexitarianism calls for an awareness of our personal impact on the world and an understanding that the morality of our diet is linked to the ecological and social conditions of human and nonhuman beings’. However, Bailey *et al*.^45^ and Laestadius *et al*.^46^ concluded from a multicountry survey, as well as interviews with non‐governmental organizations in the USA, Canada and Sweden, that, despite the clear need for tackling the demand for meat and dairy products to avoid devastating climate change, there is a remarkable lack of policies, initiatives or campaigns to do so out of the belief that it is too complex a challenge, risking alienating supporters with messages that are perceived to be negative or asking for too much. In 2018, in The Netherlands, the ‘Council for the Environment and Infrastructure’^47^ advised the Dutch Government to play an active role in reducing the national consumption of animal protein from 70% to 40% of total protein intake. The advice included setting active policy goals (e.g. setting production limits based on quotas for phosphate and CO_2_ emission, as well as number of animals), cooperating with the retail and the catering industry to stimulate innovation in the sector, as well as influence consumer behavior, educate consumers and increase the price of animal protein. However, the advice was debated by the government in March 2019^48^ and rejected; only one of six motions filed (innovation of seaweed production) was accepted.^49^ Meanwhile, based on a consumer survey in Norway, Austgulen *et al*.^50^ concluded that consumers may still not be ready to make food choices based on what is best for the climate or environment. Therefore, increased sustainability will predominantly need to come from more sustainable livestock production systems.

## HIGH INPUT–HIGH OUTPUT PRODUCTION SYSTEMS

### Improved level of outputs

A first measure towards improved output of agricultural production is optimization of the existing production process. As given by Godfray *et al*.,^51^ ‘the difference between realized productivity and the best that can be achieved using current genetic material and available technologies and management is termed the ‘yield gap’; that is, the difference between the observed yields and potential yields of crops [and livestock] at a given location.^52^ A yield gap may exist because of a mismatch between available technology, water, nutrients, land, biodiversity and labor, and their optimum use by farmers based on accessibility, market influence and knowledge.^52^ Whereas actual crop yields are already approximating their maximum possible yields in some regions, better deployment of existing crop varieties with improved management could significantly increase yields in particular across many parts of Africa, Latin America, and Eastern Europe.^52^, ^53^ The same holds true for livestock production. For example, the book by Ruth Harrison in 1964 on ‘animal machines’ was the first detailed description of livestock systems with ‘rapid turnover, high‐density stocking, a high degree of mechanization, a low labor requirement, and efficient conversion of food into saleable products’ in the Western world. In response to the rapidly growing demand for livestock products, over recent decades, large intensive livestock production units using the best genetics, in particular for pig and poultry production, have also emerged in many developing regions, closing the yield gap with respect to what is attainable in the developed world.^54^

Genetic improvement is key to this development. In the 19th Century, a combination of European and Asian pig genetics laid the foundation for the creation of modern European pig breeds, which became further genetically improved when national, regional, and commercial pig breeding companies began to develop in Europe and North America after 1945.^55^ From the late 1970s, it became common to use hybrid fattening pigs on commercial operations which improved production due to hybrid vigor and breed complementarity.^56^ Breeds used as sires could now be selected for production traits (lean growth, carcass quality and feed efficiency), whereas dam line selection also focused on reproduction traits (fertility, age at puberty, number born alive, litter weight). Worldwide, pig production is dominated by the use of the Large White, Duroc, Landrace, Hampshire, and Pietrain breeds, and breeding pigs are supplied by only few commercial companies, including Genus‐PIC, Topigs‐Norsvin, Hypor, Danbred, JSR Genetics and Choice Genetics. Selection for feed efficiency is particularly relevant to support increased production levels with fewer resources, reducing the energy needed for producing feed while reducing animal excretions. However, whereas upwards selected production traits hypothetically have no upper limit, downwards selected traits that are related to the animal's energy balance (feed intake, body fatness) do: at a value of zero. This reduction may have consequences for animal robustness.^57^

### Improving quality of inputs

Genetically improved levels of productive output on a defined limited space are likely to require increased quality, if not quantity, of inputs. Improved crop production requires irrigation, fertilizer, machinery, crop‐protection products for pest and weed control, and soil‐conservation measures.^51^ The negative externalities of production systems with high external inputs have been extensively described, for example, by Gregory *et al*.^58^ regarding losses of nutrients from fertilizers and manures to water courses and contributions of gases to climate change. This raises questions about the sustainability and potential environmental consequences of future production systems, and the need to focus on ‘sustainable intensification’; that is, ‘production methods [that] have to sustain the environment, preserve natural resources and support livelihoods of farmers and rural populations around the world’.^58^, ^59^ Sustainability can be improved by fine‐tuning the use of inputs through precision agriculture; that is, a series of technologies that allows the application of water, nutrients, and pesticides only to the places and at the times they are required.^51^

Similarly, improved levels of livestock and poultry production at high stocking densities and modern biotechnology must be supported by improved technology and increased quantity and/or quality of resources that allow for the expression of production traits. With the delinking of livestock from on‐farm, mixed‐farming agricultural by‐products, resulting from the industrialization of livestock production, feed with higher nutritional and commercial value is now sourced from international markets, including grain, oil‐meal, fish‐meal and soybean meal.^60^ Worldwide, approximately one‐third of global cereal production, 74% of maize production, and 83% of soybean production are fed to animals.^25^, ^61^ Soybean meal is a major ingredient in livestock feeds because of its relatively low water content, high protein content (approximately 40%, up to 50%) with a suitable amino acid profile, minimal variation in nutrient content, and anti‐nutritional factors that are easily reduced or eliminated, in addition to it comprising a crop that is readily available year‐round.^62^ It is a major source of the amino acid lysine, which is the first limiting amino acid for pigs and the second for poultry.^63^ Van Gelder *et al*.^64^ estimated that the soy meal content needed to produce one unit of livestock product in the EU is approximately 21 g L^−1^ for milk, 32 g egg^–1^ for eggs, 232 g kg^−1^ for beef and veal, 648 g kg^−1^ for pork and 967 g kg^−1^ for poultry meat. The share of soy production in 2014 was 31% in the USA, 31% in Brazil and 19% in Argentina.^65^ In the EU, however, grain legume crops [species of Fabaceae (Leguminosae) family, including soybean, first pea, field beans, broad beans, chick pea, lentils and lupine] are grown on only 1.8% of arable land, making Europe heavily reliant on expensive imports, comprising approximately 70% for agricultural protein products and > 95% for soybean grains and meal.^66^ The reason for Europe's low production origins from trade agreements with the USA. At the international trade negotiations of the General Tariff and Trade Agreement (GATT) Dillon Round of 1962, duty free entry of oilseeds to the European market (including soybeans) was negotiated, which, because of its significant progress in the efficiency of production and the use of new technologies, and therefore its favorable protein/cost‐ratio, left alternative European substitutes for soy unable to develop. Subsequently, with the Blair House Agreement of 1992, the USA successfully negotiated a limit to the area of subsidized oilseeds production in Europe, further increasing Europe's dependency on soy imports; this agreement was rendered obsolete with the reform of the EU's Common Agricultural Policy of 2005, which reduced oilseed payments to the same level as grains.^67^, ^68^ However, by then, the relatively few investments made in the past decades had resulted in yield gaps in developing these protein crops relative to that of wheat or maize.^69^ Imports come in particular from Argentina and Brazil,^62^ where soybean production has expanded into natural ecosystems in the Amazon (tropical forest) and Cerrado (savanna) in Brazil,^70^ and tropical dry ecosystems in Argentina.^71^ Monogastric animals such as pigs are particularly dependent on simple carbohydrates; therefore, more than 50% of their total dry‐matter intake consists of grains and 9–25% consists of oil seed cakes; soybean meal accounts for 85% of the protein supplement fed to pigs.^72^ According to Mottet *et al*.,^25^ pigs in industrial production systems consume 24.1 kg dry matter to produce 1 kg of pork protein; this 24.1 kg dry matter consists of 4.4 kg protein that originates from human‐edible sources. These pigs have a considerably better feed conversion ratio than pigs in backyard systems; however, the latter make a positive net contribution to human protein availability by producing more protein in product than the amount of human‐edible protein that they consume (0.7 kg kg^−1^ product).

### Precision livestock farming

Similar to crop production, the sustainability of this high‐input high‐output system is further improved through the use of modern monitoring and control systems that allow for precision livestock farming (PLF), a term coined in 2004 describing ‘a management system based on continuous automatic real‐time monitoring and control of production/reproduction, animal health and welfare, and the environmental impact of livestock production’.^73^, ^74^ Because feed accounts for 60 to 70% of the overall production costs of livestock production, precision livestock feeding is an important component of PLF, which consists of providing, in real‐time, the individual amount of nutrients required that maximizes nutrient utilization without loss of performance, and takes into consideration changes in nutrient requirements that occur over time and variation in nutrient requirements that exits among individual.^75^ Thus, according to PLF, dietary requirements form a dynamic process in function of the animal's own intrinsic (e.g. genetics, health, nutritional status) and extrinsic (environmental and social stressors) factors that can be monitored in real‐time.^76^ Precision feeding is accomplished through automatic measurement devices for the collection of data (e.g. feed intake, body weight, analytes), data processing and computational methods that estimate the nutrient requirements based on these inputs, and feeding systems that are capable of providing (individually) the adequate amount and precise diet formulation that maximize the desired production trajectory.^75^ For example, by modeling the individual nutrient requirements of sows and growing pigs, feeding strategies can be formulated on a per animal basis, thus optimizing efficiency and performance.^74^, ^77^ Because population feed requirements in commercial pig farming are often tailored to the most demanding pigs in order to maximize the desired population response, precision feeding will prevent pigs from receiving more nutrients than they need. This improves the efficiency of dietary nutrient utilization, reduces feeding costs, and reduces environmental consequences of the excretion of excess nutrients such as nitrogen and phosphorus.^78^ As reviewed by Neethirajan,^79^ model input biomarkers that can be monitored on an animal include blood parameters, sweat and saliva sensing (e.g. for analytes including sodium, potassium, lactate, glucose, cortisol content or amounts of active drugs), body temperature, behavior and movement, stress, sound, pH, and the presence of viruses and pathogens. When this information is integrated into a monitoring system, it allows for the production of an accurate real‐time health status and disease diagnosis, keeping livestock production one step ahead of invisible diseases.^79^ The use of behavior detection (e.g. changes in feeding and drinking behavior, elimination behaviors, social behaviors and locomotion and posture) in monitoring of health and welfare is extensively reviewed by Matthews *et al*.^80^ According to Tedeschi and Menendez,^81^ mathematical modelling of decision support systems is more important than ever because it gives the user the ability to quickly evaluate multiple scenarios of production, which minimizes risks and maximizes profits, improving the acceptability, sustainability and resilience of animal production systems.

## REDUCED INPUT–REDUCED OUTPUT PRODUCTION SYSTEMS

As discussed by Van Grinsven *et al*.,^82^ when sustainable intensification still translates to intensification of land use, increasing external inputs, and the use of high yielding crop and animal varieties, this may be regarded as unsustainable in view of risks for the environment, especially in regions with a small yield gap, such as in Europe. In addition, several studies suggest that animals in high‐density stocking with genetically high levels of production, and depending on advanced animal nutrition and animal management practices to support their productive potential, are more sensitive to changes in the production environment. For example, a simulation study by Kolmodin *et al*.^83^ suggested that environmental sensitivity will increase with selection for high phenotypic production values. These observations were supported by Knap and Su,^84^ who indicated that ‘irrespective of genetic effects, the performance of sows with a high reproductive capacity is practically always highly sensitive to environmental disturbance (…) the performance of high‐potential genotypes (and of high‐capacity sows) will likely come down strongly when environmental conditions become unfavourable’. This is relevant because livestock animals are required to perform in a wide variety of environmental, often suboptimal, conditions.^22^, ^23^

### Climate change

**Figure 3:**
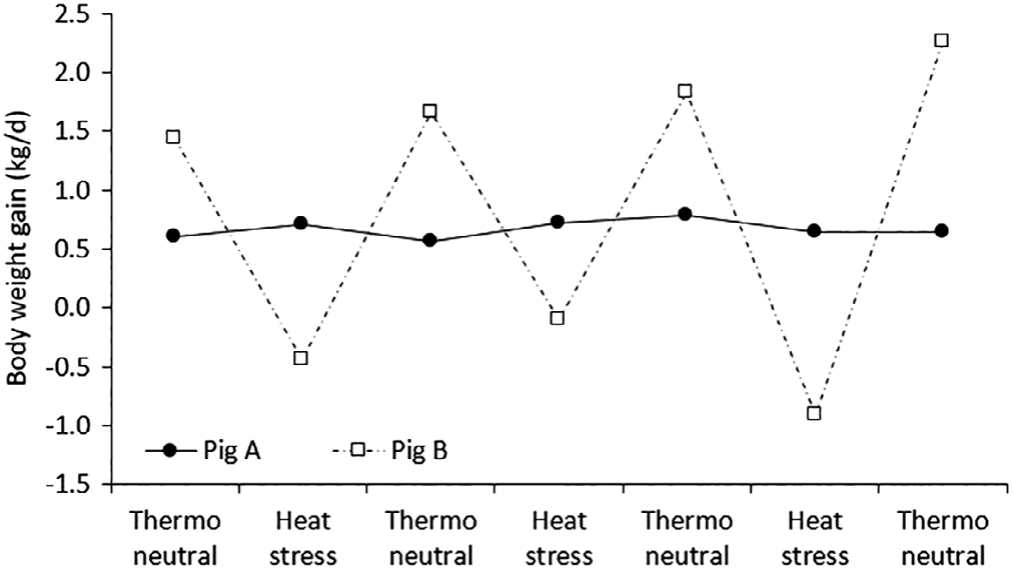
Body weight gain (BWG) of two extreme examples of individual observations on pigs A and B that depict the negative correlation between BWG in a thermoneutral environment and that during heat stress. After Rauw *et al*.^89^

For example, a shift towards warmer climates may move livestock animals out of their thermal comfort zones, resulting in reduced feed intakes. Furthermore, drought and extreme rainfall variability, as well as other smaller climatic changes, can trigger periods of feed scarcity and changes in the nutritional quality of feeds.^85^ Genotype by environment interactions have been particularly well described in dairy cattle, where high genetic potential cows that are transferred to tropical environments may lose their superiority in production.^86^ High potential Holstein dairy cattle ate more and had higher growth rates under low environmental stress conditions (no parasites or diseases, no heat stress, and a high quality diet) than low potential Brahmans, whereas Brahmans had the highest realized growth of the two breeds at high levels of environmental stress.^87^ Poullet *et al*.^88^ showed that Creole pigs, which are adapted to tropical conditions, were able to maintain body weight gain under restricted feeding (a common physiological response of growing pigs facing stressful environmental conditions such as heat stress), whereas growth performance in Large White pigs, which are selected for high production performance, was significantly reduced. Similarly, Rauw *et al*.^89^ observed that, independent of genetic line, pigs with higher growth rates in a thermoneutral environment had lower growth rates in a subsequent heat stress challenge, indicating that high producing animals in thermoneutral conditions were less robust to heat stress, whereas those robust to heat stress showed a trade‐off with production under thermoneutral conditions (Fig. 3). This is also supported by observations by Settar *et al*.,^90^ who reported that broiler genotypes that gain more weight in the spring tended to gain less weight under the hot conditions of summer. Rauw *et al*.^89^ concluded that the results of their study emphasize the need to review breed choice and genetic selection objectives for improved heat tolerance to climate change. Pigs of interest as selection candidates are those that are able to maintain high growth rates under heat stress, and these animals may not have the genetics with highest growth potential.

### Alternative feed resources

Also, the notion of feeds with high nutritional value sourced from international markets as high inputs to support the high production potential of genetically improved livestock as a solution to the sustainability issue is being challenged. According to Van Zanten *et al*.,^91^ it is increasingly recognized that we might better not use highly productive croplands to produce human edible crops such as cereals to produce feed for livestock. High‐quality feeds may involve large losses of potential human‐edible food in their production. Cassidy *et al*.^61^ found that, in 41 crops analyzed in their study, 36% of the 9.46 × 1015 cal available in plant form go to animal feed. Of this 36%, 89% is lost, such that only 4% of the calories in animal feed crops ultimately contributes to the human diet in the form of animal products.^61^ According to Rifkin^92^ grain‐fed cattle, pigs and chickens are consumed mostly by affluent populations, especially in Europe, North America and Japan, calling it ‘a new form of human evil, with consequences possibly far greater and longer lasting than any past wrongdoing inflicted by men against their fellow human beings’. Instead, feeding co‐products from human food, food waste and biomass from marginal lands will contribute to sustainable nutrition security and maximize the number of humans that can be fed per hectare.^91^ Zu Ermgassen *et al*.^93^ estimated that 1.8 million hectares of agricultural land could be saved if EU legislation would change to allow the use of food wastes as animal feed, which is currently illegal for most wastes because of disease control concerns. In a follow‐up study, Salemdeeb *et al*.^94^ found that converting municipal food wastes into pig feed would lead to lower environmental and health impacts than processing waste by composting or anaerobic digestion. However, in addition, the notion of unsustainability of the heavy dependency of the EU to soybean meal imports at the mercy of price volatility of international markets resulted in a motion for a resolution in the European parliament that was adopted on the 1^st^ of January 2011, emphasizing the potential to make the supply of animal feed more reliable by use of agro‐environmental measures. This includes growing on‐farm animal feed using mixed crops such as cereals and beans, encouraging extended crop rotation systems that integrate protein crop production into the system, using by‐products of oilseed and agrofuels production for animal feed, and providing adequate financial support to farmers involved in sustainable or organic agricultural production.^95^ In 2013, a Focus Group on Protein Crops involving 20 experts from 11 EU countries, set up by the European Innovation Partnership in Agriculture, came together to discuss the question ‘How can the competitiveness of protein crops producers in the EU be improved?’. The group analyzed the potential to increase productivity and protein content of soybeans, rapeseed, sunflower, lupin, pea, faba beans, alfalfa and clover^63^ and the results were published in their final report.^69^ The central conclusion to the data is that protein crops have a long way to go before being competitive, although this can be stimulated through different aspects of innovation, including technical innovations on agronomy (variety choice, fertilization, disease control, water use, crop mixtures, environmental effects and rotational aspects) and breeding (focusing on drought resistance, climate adaptability, disease resistance, protein content and reduction in anti‐nutritional factors).^63^, ^69^

However, because the quantity and quality of feed resources limits productive output, feeding diets of suboptimal nutritional quality may result in genotype by diet interaction. Indeed, Brandt *et al*.^96^ observed a clear genotype environment interaction for growth in different pig breeds kept under conventional (i.e. standardized diets of the performance testing station) and organic production systems (organic diets based on farm‐grown feedstuffs). However, genotype by diet interactions may also occur when the same genotypes are offered different diets or different quantities of the same diet. Mauch *et al*.^97^ observed that responses to selection for improved feed efficiency when fed higher‐energy, lower‐fiber diets was not fully realized when pigs were instead fed an extremely lower‐energy, higher‐fiber diet. Rauw *et al*.^98^ observed that pigs that were more feed efficient on a high quality concentrate diet were less feed efficient on a high‐fiber local diet. These observations emphasize that, when sustainability of production is enhanced by improving the efficiency of pigs to transform local, low quality feed into meat,^99^ this may require a different type of pig than those currently selected in intensive, high quality input–high output production systems. As reviewed by Phocas *et al*.,^100^ the agroecological management of pigs, which includes decreased external inputs needed for production and decreased pollution by optimizing the metabolic functioning of farming systems, calls for animals with different performance characteristics and the need to breed for robustness across environments.

## PROSPECTS FOR SUSTAINABLE PIG PRODUCTION

This perspective has discussed two alternative scenarios for improving the sustainability of future pig production systems while aiming at feeding 11 billion people by 2100. The first scenario, a high input–high output scenario, is based on sustainable intensification, maximizing animal protein production efficiency on a limited land surface while minimizing environmental impacts. This is accomplished through precision livestock farming, using animals selected for highest production potential (animal Type B in Fig. 3) that are precisely fed with genetically improved (imported) crops produced via improved production methods, and that are monitored for disease and welfare issues. The second scenario to sustainable pig production, a reduced input–reduced output scenario, is based on selecting animals that are more robust to climate change and are better adapted to transform low quality feeds (local feeds, feedstuff co‐products, food waste) into meat (animal Type A in Fig. 3). However, similar to organic farming,^101^ the feasibility of this latter scenario may be contested not only because of reduced predicted yields and reduced production efficiency, but also because of higher costs as a result of the reorganization of the feed supply chains, the need to supplement for unbalanced nutrient quality or the pre‐treatment of feedstuffs to, for example, reduce anti‐nutritional factors.^102^

National evaluation of the availability of local feed and feedstuff co‐product alternatives is a first step to evaluate the feasibility of the reduced input‐reduced output scenario. For example, the Foods of Norway initiative at the Centre for Research‐based Innovation in Ås, Norway,^103^ has been set up with the specific aim to move Norwegian livestock production away from importing plant ingredients such as soy, and developing with novel technology novel feed ingredients from local natural bioresources. Alternative feed ingredients investigated include yeast derived from spruce trees, macroalgea, rapeseed, and co‐products from fish, animals and plants,^104^, ^105^, ^106^, ^107^. Industrial partners,^108^ as well as the Norwegian Ministry of Agriculture and Food,^109^ are highly supportive of the initiative. Subsequently, modelling is required to evaluate the implications of different scenarios. For example, Röös *et al*.^27^ evaluated the implications in Western Europe for land requirement and environmental consequences of livestock intensification, assuming closure of crop yield gaps, increased livestock production efficiencies and reduced waste at all stages for different food consumption scenarios. They conclude that land use and greenhouse gas emissions could in principle be halved; however, it would still not be sufficient to reach EU climate change targets.^27^ In dairy production, an integrated farm system model software tool was developed to assess and compare the environmental and economic sustainability of farming systems based on nutrient flows from crop production, feed allocation, production responses and manure production to predicted losses to the environment.^110^ Several studies also modelled the implications for switching to local feedstuffs. For example, Life Cycle Assessment (LCA) by Sasu‐Boakye *et al*.^111^ predicted that, in Sweden, local protein feed production will present an opportunity to reduce greenhouse gas emissions but at a cost of increasing land occupation for feed production. Depperman *et al*.^102^ assessed the market impacts of a complete switch to regionally produced feed in the European livestock sector. They predicted that an implementation would cause a significant increase in the costs of livestock production, which may be counteracted when this is combined with a reduction in consumption of livestock products. The Global Feed Lifecycle Assessment Institute, an independent feed industry initiative launched in 2016, develops a freely and publicly available global cradle to farm‐gate LCA database and tool for the evaluation of feed industry environmental impacts.^112^ In addition, Ottosen *et al*.^113^ developed a method for estimating the environmental impact from (correlated) genetic change in intensive pig production systems. Their study showed that finisher growth rate, body protein‐to‐lipid ratio, and energy maintenance could be important in reducing environmental impacts, but mortalities and sow robustness had little effect. Furthermore, Zira *et al*.^114^ developed a social LCA model with an analytical hierarchical processing method for prioritizing low social sustainability antecedents of poor conditions for farm workers and animals in pig production. Based on a pilot study, the highest priority for worker issues should be given to income; for pig issues, similar priority should be given to health, ambient temperature, handling at slaughter and freedom to exhibit natural behavior. In a systematic review of LCAs, McClelland *et al*.^115^ warn that simplified LCAs that focus on a single impact category alone may result in risk misinterpretation and misrepresentation of the full extent of livestock production impacts on the environment.

Given a predicted reduction in yields and production efficiency, and an increase in costs of the reduced input‐reduced output scenario, it may be deducted that the high input–high output scenario of intensification of livestock production is more suitable when the aim is to increase the amount of animal protein products. However, the need for intensification towards increased output of agricultural production is challenged by Holt‐Giménez *et al*.^116^: ‘We already grow enough food for 10 billion people … and still can't end hunger’. They state: ‘Hunger is caused by poverty and inequality, not scarcity. For the past two decades, the rate of global food production has increased faster than the rate of global population growth’, producing already enough to feed the world's 2050 projected population of 10 billion people as long as the bulk is not diverted to the production of biofuels and to feed confined animals. Foley^117^ estimates that up to three quadrillion additional calories can be added to the food supply, some 50% from our current supply, if humans would switch to all‐plant diets. Although the overall demand for animal products is increasing at a rate that may be underestimated,^118^ the overreliance on animal‐based foods as a source of protein has steadily decreased in the developed world, resulting in a steady increase in the number of vegans, vegetarians or flexitarians who focus on the health benefits of a meat‐free diet or are concerned by the treatment of confined livestock and the negative implications of livestock production for our environment.^119^ Between 2014 and 2017, consumers following a low‐meat diet increased from 26% to 44% in Germany, whereas consumers claiming to be vegan increased from 1% to 6% in the US.^120^ According to a modelling study by Westbroek *et al*.,^121^ replacing just 50% of animal‐derived foods (meat, dairy products, and eggs) with plant‐based foods in the European Union would result in a 40% reduction in nitrogen emissions, a 25–40% reduction in greenhouse gas emissions, a 23% *per capita* less use of cropland for food production and a 75% reduction of the use of soymeal, whereas dietary changes would lower our health risks. In addition, an estimated one‐third of all food produced globally is either lost in the supply chain or wasted, with the latter including food that deviates from what is considered the correct shape, size or color, food that is close to or beyond the ‘best‐before’ date, and food that is disposed of at households and restaurants.^122^ FAO's Target 12.3, as specified under the ‘Sustainable Development Goals’, aims ‘by 2030 to halve per capita global food waste at the retail and consumer levels and reduce food losses along production and supply chains, including post‐harvest losses’.^123^ Therefore, a reduced input‐reduced output scenario is feasible if this is combined with a reduction in meat consumption and food waste, which was urgently called for in August 2019 by the Intergovernmental Panel on Climate Change to help fight climate change.^124^

Furthermore, in the reduced input‐reduced output scenario, a necessary condition to maintain farm income on potentially reduced production yields and reduced production efficiency is compensation through price premiums on the products and/or savings on external inputs.^82^ Napolitano *et al*.^125^ reviewed several willingness‐to‐pay studies and concluded that people express interest to pay a premium for food from animals raised humanely. With this public endorsement the European Union has been able to increase the number of regulations on the welfare of farm animals over the past years.^125^ Maynard *et al*.^126^ observed that a considerable proportion of consumers are also willing to pay premiums for meats sold under a ‘locally produced’ label. A recent study by Profeta and Hamm^127^ showed that German consumers’ willingness to pay a premium for animal products produced with local feeds may account for the higher prices of such products when they are sold as a differentiated product in local supply chains. However, extensive consumer communication is necessary to raise awareness on feed origin and feed imports for animal production.^127^ In addition, although the willingness to replace meat with meat substitutes, insects or cultured meat is currently low,^128^ they offer a potential sustainable alternative to livestock protein production.^129^ For example, according to Van Huis and Oonincx,^130^ environmental advantages of insect farming compared to livestock production are lower requirements for land and water, lower greenhouse gas emissions, better feed conversion efficiencies, and the ability to transform low‐value organic by‐products into high‐quality food or feed. Once the infrastructure for production, processing, storage, distribution and marketing, as well as the legislation for their use, is realized, this will offer a tremendous potential for cheap mass production of protein.^131^ The fast‐food industry has been and remains one of the major catalysts for cheap meat production.^132^ Given these future novel sustainable alternatives to livestock animal protein, it may become reasonable to move towards an added general premium price or taxation on ‘protein from livestock animals’ to the benefit of promoting higher incomes to farmers at the same time as covering the extra costs of (politically enforced) welfare of livestock animals in sustainable production systems, to the benefit of animal production in both scenarios. In 2016, the Danish Council on Ethics proposed taxation on meat considering that consumers should make an ethical commitment to take the implications for the climate of our eating habits into account.^133^ Such developments may be right around the corner, as exemplified by the resolution proposed by the Dutch political ‘Party for Animals’ in October 2019 to increase taxation of animal slaughter with the aim to reduce meat consumption.^134^

Eventually, determination of limits to feed sourced from international markets, the availability of local feed and feedstuff co‐product alternatives, available land for crop and livestock production, and desired production levels, together with a willingness to politically enforce policies through subsidies and/or penalties, are some of the considerations that must be taken into account with respect to the development of new pig production systems.

## SYNTHESIS

The human population is projected to rise to almost 11 billion people by 2100. Parallel to population growth, there is evidence for an increase in meat production and consumption. Livestock farming systems provide a large range of benefits, including food security, employment and ecosystem services. Meat supplies energy, protein and important micronutrients. However, despite these multiple benefits of meat production, an increase in the number of livestock animals directly challenges sustainability of animal production, through increased requirements for land, water and energy, as well as increased anthropogenic emissions of greenhouse gasses and waste. Sustainability of pig production can be improved through precision farming techniques in high input–high output production systems, providing, through the use of an automatic real‐time management system, genetically improved individual animals with exactly the amount of high‐quality resources required for maximum production efficiency, minimizing losses and waste. For example, precision feeding will improve the efficiency of dietary nutrient utilization, and therefore reduce environmental consequences of the excretion of excess nutrients. However, technification of livestock systems is not available to the entire livestock sector. Instead, modern livestock animals are often challenged to perform in a wide variety of suboptimal environmental conditions; for example, regarding climate and differences in feed quality and composition. In addition, the EU is interested in stimulating on‐farm animal feed production to reduce its heavy dependency on imported feedstuffs such as soybean meal. Therefore, sustainability of pig production can be improved selecting pigs with higher tolerance to climate change and with a shift from reliance on optimally formulated feeds to local feeds and feedstuff co‐products of sub‐optimal quality. Although the economic feasibility of this reduced input‐reduced output scenario may be contested because of reduced predicted yields, reduced production efficiency and higher costs, recent technological and societal developments in food production and consumption patterns may open up new opportunities. First, the increased concern of consumers regarding the treatment of confined livestock, the health concerns of high meat intake and the negative implications of livestock production for the environment is resulting in a steady increase in the number of vegans, vegetarians or flexitarians, resulting in reduced meat consumption *per capita* in parts of the world. In addition, the FAO aims by 2030 to halve *per capita* global food waste at the retail and consumer levels and reduce food losses along production and supply chains, increasing food availability to feed the increase in human population. Second, willingness‐to‐pay studies suggest consumers’ willingness to pay a premium for animal products produced with local feeds, which may account for the higher prices of such products when they are sold as a differentiated product. Finally, recent developments in the production of meat substitutes offer the potential for cheap mass production of protein, demonstrating the opportunity to add a premium price or taxation on ‘protein from livestock animals’ to the benefit of promoting higher incomes to farmers at the same time as covering increased costs of sustainable production systems. Evaluation of the availability of and limits to production resources, together with willingness to politically enforce policies, may result in the design of new pig farming systems in which both production scenarios can co‐exist.

## CONFLICT OF INTERESTS

The authors declare that they have no conflicts of interest.
